# Role of Serotonin in Cadmium Mitigation in Plants

**DOI:** 10.3390/plants14121738

**Published:** 2025-06-06

**Authors:** Hesham F. Oraby, Nehal Z. Elnaggar, Ahmad A. Omar, Azza H. Mohamed

**Affiliations:** 1 Department of Crop Science, Faculty of Agriculture, Zagazig University, Zagazig 44519, Egypt; heshamoraby@gmail.com (H.F.O.); nehal.elnaggar282@yahoo.com (N.Z.E.); 2 Biochemistry Department, Faculty of Agriculture, Zagazig University, Zagazig 44511, Egypt; 3 Citrus Research and Education Center, University of Florida (IFAS), Lake Alfred, FL 33850, USA; azza@ufl.edu; 4 Department of Agricultural Chemistry, College of Agriculture, Mansoura University, Mansoura 35516, Egypt

**Keywords:** cadmium toxicity, tolerance mechanisms, serotonin, stress response, reactive oxygen species

## Abstract

Contamination of the soil with cadmium (Cd) presents serious hazards to plant growth, ecosystem harmony, and human health. Plants have evolved various mechanisms to address Cd toxicity, such as sequestration, chelation, and antioxidant defense systems. Knowledge of these mechanisms is an important requisite for the development of strategies to relieve Cd stress in plants. More recent studies also implicate the role of the neurotransmitter 5-hydroxytryptamine (serotonin) in enabling Cd mitigation behavior in plants. Beyond its well-known role in animals, serotonin has emerged as a vital signaling molecule in plants, contributing to stress responses and regulatory pathways. This review focuses on the different Cd tolerance mechanisms in plants and describes the role of serotonin in protection against Cd toxicity. Moreover, it investigates how serotonin interacts with other signaling molecules to coordinate Cd stress responses. Understanding the intricate network of Cd tolerance mechanisms and the involvement of serotonin is essential for developing effective strategies to combat Cd stress in plants and improve environmental quality.

## 1. The Effect of Cadmium and Its Toxicity in Plants

Due to the industrial revolution, plants’ exposure to agricultural soils contaminated with different heavy metals has substantially increased, endangering biota and disrupting the food chain. Even trace concentrations of non-essential heavy metals such as lead (Pb), mercury (Hg), arsenic (As), and cadmium (Cd) are toxic to living organisms [1,2,3].

Cadmium (Cd) is a toxic heavy metal that can have detrimental consequences for plants when present in excessive quantities in the soil or water. Its toxicity comes from several mechanisms that disrupt various physiological processes within plant cells [4,5]. Studies have revealed that it has damaging consequences for germination and the vegetative and reproductive stages due to anatomical, morphological, and biochemical changes such as alterations in the photosynthetic machinery and membrane permeability [4]. Cadmium interferes with and disrupts various stages of photosynthesis, including light absorption, electron transport, and carbon fixation [6]. It directly affects energy utilization, carbon sequestration, and different enzymes involved in photosynthesis, such as ribulose-1,5-bisphosphate (RuBP) carboxylase oxygenase, phosphoenolpyruvate carboxylase, aldolase, fructose-6-phosphate kinase, fructose-1,6-bisphosphatase, NADP+-glyceraldehyde-3-phosphate dehydrogenase, and carbonic anhydrase [7,8,9,10]. Cd exposure inhibits the Calvin cycle and other carbon assimilation reactions. In parallel, it causes chlorosis by disturbing chlorophyll biosynthesis, thereby impairing light harvesting in the thylakoids [11]. Cd ions can replace magnesium (Mg) in the chlorophyll molecule, disrupting its structure and function. This inhibition of photosynthesis results in reduced carbon assimilation, decreased biomass production, stunted growth, and ultimately diminished yield potential [12,13].

A primary mechanism through which cadmium exerts its toxicity is inducing oxidative stress in plant cells. Cd ions promote the production of reactive oxygen species (ROS) such as superoxide radicals (O_2_•−), hydrogen peroxide (H_2_O_2_), and hydroxyl radicals (•OH) through redox reactions with cellular components [14]. These ROS can cause oxidative damage to lipids, proteins, nucleic acids, and other cellular components, interrupting membrane integrity, enzyme function, and DNA stability. This causes an imbalance between the production of reactive oxygen species (ROS) and the capacity of the antioxidant defense system to detoxify them and consequently cell death [15].

Also, Cd-induced lipid peroxidation compromises membrane integrity and fluidity, affecting ion homeostasis and nutrient uptake [16]. It causes the leakage of cellular contents and a loss of compartmentalization [17]. Furthermore, the accumulation of cadmium in organelles such as chloroplasts, mitochondria, and the endoplasmic reticulum (ER) alters their structure and function, impairing cellular metabolism and energy production. In chloroplasts, cadmium inhibits photosynthetic electron transport and damages thylakoid membranes, impairing ATP, and NADPH synthesis [18]. Similarly, cadmium impairs mitochondrial electron transport, diminishing ATP production and augmenting ROS generation [19]. The endoplasmic reticulum, involved in protein folding and lipid metabolism, is also affected, leading to protein misfolding and ER stress [20]. It induces the unfolded protein response (UPR) primarily throughout the bZIP60 arm, which in turn triggers significant endoplasmic reticulum stress marker genes like BiP3, CNX, PDI5, and ERdj3B in a time- and concentration-dependent manner [20].

Furthermore, cadmium competes with essential mineral nutrients such as zinc (Zn), iron (Fe), calcium (Ca), and magnesium (Mg) for uptake by plant roots [21,22]. Excessive cadmium accumulation can restrict the uptake, transport, and assimilation of these nutrients within the tissues, causing nutrient imbalances and plant deficiencies [23]. For example, cadmium can replace zinc in enzymes such as carbonic anhydrase, affecting their activity. This disturbance in mineral nutrient uptake and utilization weakens various metabolic and physiological processes, including enzyme catalysis, osmotic regulation, hormone synthesis, and cell signaling [24,25,26]. When cadmium interferes with the signal transduction pathways involved in plant growth, development, and stress responses, it hinders calcium (Ca^2+^) signaling, which regulates numerous physiological processes, including stomatal closure, gene expression, and hormone signaling [27]. Cadmium also affects plants’ stomatal regulation, transpiration rates, and water uptake and balance. Exposure to Cd can induce closure of the stomata, limiting gas exchange and reducing photosynthetic activity and transpiration rates. This closure helps conserve water but can also lead to decreased nutrient uptake due to reduced movement of water through the roots [28,29]. Additionally, cadmium-induced oxidative stress can damage the root tissues, causing water stress, which exacerbates the detrimental effects of cadmium toxicity, further impairing plants’ growth and productivity [28].

Cadmium has genotoxic effects on plant cells regarding genetic materials, causing DNA damage, chromosomal aberrations, and mutations [30]. It can directly interact with DNA molecules, leading to DNA strand breaks, base modifications, and cross-linking. This genotoxicity results in heritable changes in the plant genome, affecting growth, development, and reproductive fitness. DNA damage triggers cell cycle arrest or programmed cell death in affected plant tissues [31].

**Figure 1:**
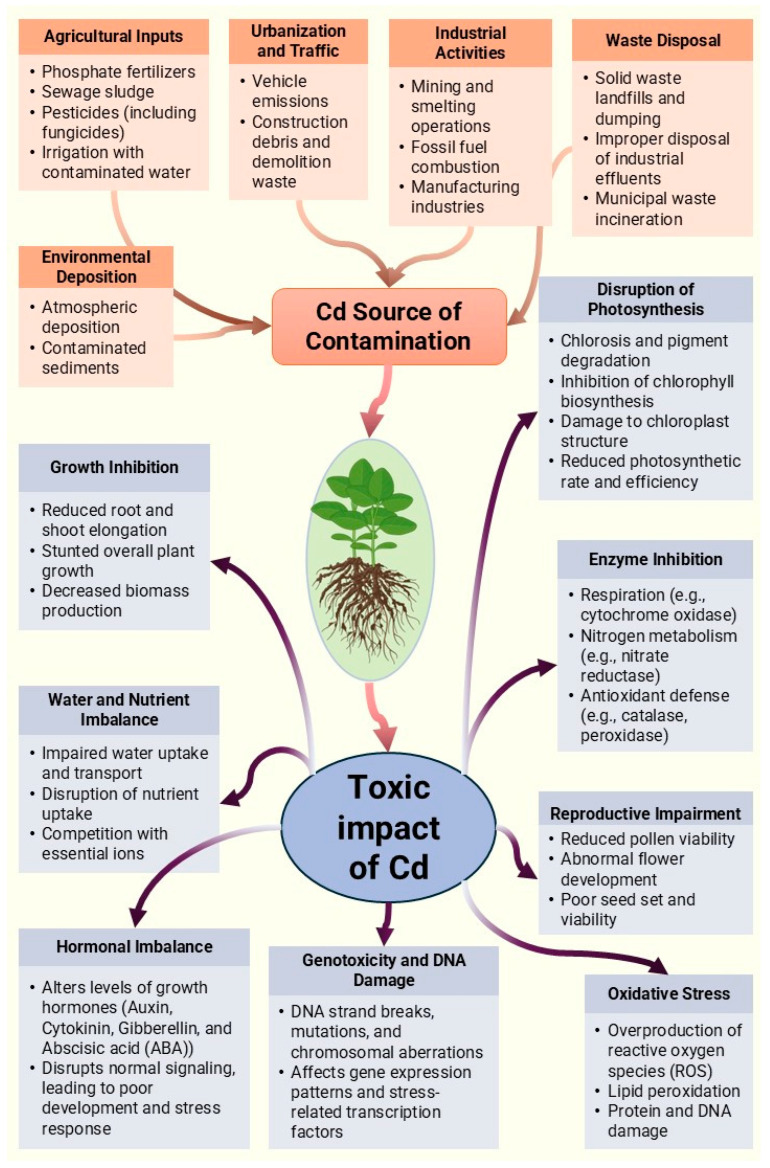
The potential sources of Cd contamination and its more toxic impacts on plant health.

Understanding the mechanisms underlying cadmium toxicity is essential for developing strategies to mitigate its impact on plants’ health and productivity and ensure food security and environmental sustainability. Figure 1 summarizes the potential sources of Cd contamination and its more toxic impacts on plant health.

## 2. Cadmium Tolerance Mechanisms

Cadmium exposure triggers significant changes in gene expression patterns in plants. Transcriptomic analyses have revealed alterations in the gene expression in various cellular processes, including stress response, metal transport and sequestration, antioxidant defense, and detoxification pathways [32].

The buildup and transfer of Cd in the aerial portion of plants have been linked to a number of mechanisms. The physiological processes that cause cadmium to accumulate in the plant system include binding to the root cell walls and sequestration in the root vacuoles and xylem tissue [33]. The distribution of Cd within the cells reveals that it mostly accumulates in the cell wall, with a soluble fraction found in organelles and membranes in the tissues of the roots and leaves [34,35]. The first line of defense against stress is the cell wall, which is made partly of pectin. Increased Cd accumulation in the cell wall is caused by an increased concentration of negatively charged groups in the pectin components, which positively interact with Cd [36]. Cadmium (Cd) detoxification through nutrient supplementation has been widely reported as an effective strategy for mitigating its toxic effects in plants. It enhances antioxidant defenses and promotes the synthesis of metal-chelating compounds such as phytochelatins. For example, silicon has been shown to reduce the translocation of Cd from the roots to the shoots and boost plant resistance by strengthening cell walls [37]. Also, foliar choline spraying before or after Cd exposure significantly decreased the Cd uptake in Solanum lycopersicum seedlings, improving growth and antioxidant defense systems [38]. Similarly, higher zinc concentrations in hydroponic solutions reduced the absorption of Cd in lettuce (*Lactuca sativa*), resulting in better growth and fewer indicators of oxidative stress [39].

Plants enhance several enzymatic and non-enzymatic antioxidant activities to prevent oxidative molecules such as reactive oxygen species (ROS) from damaging their cell membranes, including the plasma membrane [40]. Higher levels of glutathione reductase, catalase, ascorbate peroxidase, and superoxide dismutase have been shown to improve plants’ tolerance to Cd stress [41,42,43]. Phenylalanine ammonialyase (PAL), peroxidase (POD), 4-coumarate CoA ligase (4CL), caffeic acid 3-O-methyl transferase (COMT), cinnamyl alcohol dehydrogenase (CAD), and cafeoy1-CoA3-O-methy1transferase (CCoAOMT) are among the enzymes that catalyze the phenylpropanoid pathway. The phenylpropanoid pathway is generally connected with the production of flavonoids, monolignols, phenolic acids, stilbenes, and coumarins, and these compounds induce a stress response in plants [44,45].

Plants have established sophisticated mechanisms to detoxify and sequester cadmium ions. Phytochelatins (PCs) and metallothioneins (MTs) are small, cysteine-rich peptides that bind to cadmium ions and form complexes, which are then sequestered in the vacuole, thus reducing cytosolic cadmium concentrations [46]. Vacuoles are considered the cell organelle that plays the central part in Cd retention. Two cassette transporters (AtABCC1 and AtABCC2) control the method through which the metal–phytochelatin (PC) complex is contained in the vacuole [47]. Furthermore, the activation of multiple important genes linked to metal transporters, chelator proteins, antioxidant enzymes, defense genes, and transcription factors in plants is necessary for enhanced Cd tolerance [48]. Through the action of several transporters, cadmium can migrate from the soil and accumulate in the consumable parts of plants. Disruption of these transporters could be a useful strategy to combat Cd accumulation. One member of these transporter families is heavy metal ATPase (HMA), often called P-type ATPases, which can absorb and transport heavy metal ions, including Cd [49]. Similarly, iron-regulated transporter (IRT1), also known as ZRT/IRT-like proteins, is one of the ZIP family members involved in Cd^2+^ transport. The Natural Resistance-Associated Macrophage Proteins (NRAMPS), a family of proton/metal transporter proteins, are in charge of the uptake of the nutritionally essential divalent cations Fe^2+^, Mn^2+^, Zn^2+^, and Cd^2+^. The functional expression of this gene in yeast indicated that it could be targeted for genetic selection or modification [50]. Furthermore, the Cd–chelate complex crossing the tonoplast is transported with the assistance of cation exchangers (CAX) [33], and environmental Cd stress signaling involves the SNF1-related protein kinase 2 subfamily protein (SnRK) [51,52]. Through controlling functional gene expression, transcription factors (TFs) such as MYB, WRKY, C2H2, bZIP, AP2, ERF, and DREB also significantly contribute to the tolerance to metal stress in a range of plants [53,54,55].

**Figure 2:**
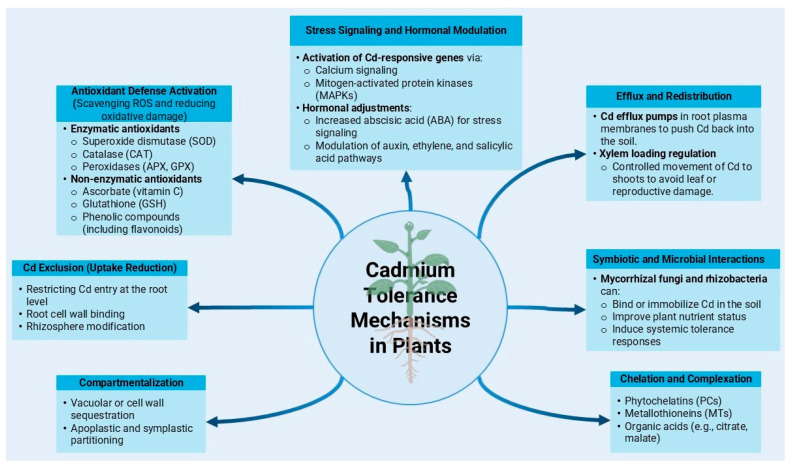
Several possible mechanisms of cadmium tolerance in plants.

There is considerable genetic variability among plant species and cultivars in terms of their tolerance to cadmium toxicity. Some plant species, known as hyperaccumulators, have evolved mechanisms [56] for tolerating and accumulating elevated levels of cadmium in their tissues without experiencing toxic effects [56]. Understanding the genetic basis of cadmium tolerance and hyperaccumulation can aid in identifying and breeding crop varieties tolerant to cadmium-contaminated soils. Figure 2 illustrates several possible mechanisms of cadmium tolerance in plants.

## 3. Serotonin Biosynthesis

Serotonin (Ser), also known as 5-hydroxytryptamine (5-HT), is a neurotransmitter and signaling molecule with diverse functions, especially in the physiological processes of both animals and plants [57]. Serotonin serves as one of the controlling regulators of plant growth and development by affecting various cellular processes. In plants, serotonin plays roles in growth, development, and defense mechanisms as part of the plants’ stress response [58]. Its biosynthesis and catabolism refer to the processes by which serotonin is produced and broken down within the cells.

Serotonin biosynthesis involves a sequence of enzymatic reactions that convert precursor molecules into serotonin [59,60]. The pathway for serotonin biosynthesis in plants typically begins with the amino acid tryptophan, an essential amino acid synthesized de novo via the shikimate pathway [61]. Regulation of tryptophan’s availability can influence plants’ serotonin levels, highlighting the importance of tryptophan metabolism as a precursor in serotonin biosynthesis [62].

The initial dedicated step in serotonin biosynthesis is the decarboxylation of tryptophan to form tryptamine. This reaction is catalyzed by the enzyme tryptophan decarboxylase (TDC), which removes the carboxyl group (-COOH) from tryptophan [63]. TDC is a pyridoxal phosphate (PLP)-dependent enzyme, and the expression of TDC genes can be regulated by various internal and external factors, such as light, hormones, and stress conditions [64].

Following the formation of tryptamine, the next step is hydroxylation at the 5-position to form serotonin. This reaction is catalyzed by the enzyme tryptamine 5-hydroxylase (T5H), which adds a hydroxyl group (-OH) to tryptamine. T5H is a cytochrome P450 monooxygenase involved in synthesizing various indole alkaloids in plants and requires molecular oxygen and NADPH as cofactors for its activity [65]. The expression and activity of T5H can also be regulated by various factors, including the developmental stage, environmental conditions, and signaling molecules [65]. It is worth mentioning that serotonin biosynthesis competes for the same tryptophan pool with the production of indoleacetic acid (IAA), the main auxin hormone in plants.

The serotonin produced in plants can undergo acylation, a process in which a fatty acid molecule is attached to serotonin. This modification is catalyzed by acyl-CoA-dependent serotonin N-acyltransferase (SNAT) to form *N*-acetylserotonin, which is then methoxylated by *N*-acetylserotonin methyltransferase (ASMT) to form melatonin. The addition of fatty acids to serotonin can alter its properties, such as its solubility, stability, and cellular localization. The *N*-acetylserotonin derivatives generated through this process may have specific functions in plant physiology, although their precise roles are still being elucidated [65,66].

In addition to acylation, serotonin in plants can undergo further modifications such as methylation and glycosylation. These modifications can form various serotonin derivatives with distinct chemical properties and biological activities [67]. For example, the methylation of serotonin can affect its stability and activity, while glycosylation can influence its solubility and cellular localization. These modified serotonin derivatives may have specific functions in plant physiology, including the regulation of growth, development, and responses to environmental stresses [68]. Understanding the regulation of serotonin biosynthesis and the functions of serotonin derivatives in plants is crucial for unraveling the complex networks underlying plant physiology and stress responses. It can provide insights into potential applications in agriculture, such as enhancing crops’ stress tolerance.

## 4. Serotonin Acts as a Master Regulator in Abiotic Stress in Plants

**Figure 3:**
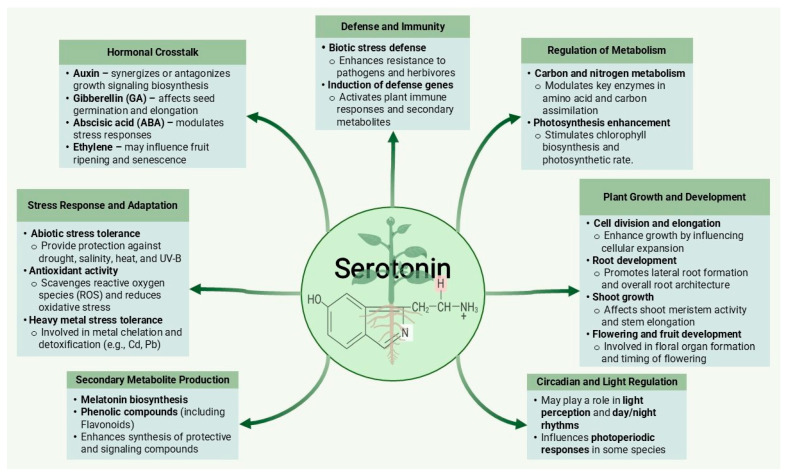
Serotonin’s functions in plants.

Serotonin plays a central role in orchestrating and coordinating plants’ responses to several abiotic environmental stressors, such as drought, salinity, extreme temperatures, and heavy metal toxicity (Figure 3). As a signaling molecule, serotonin serves as a principal regulator that integrates with and modulates key signaling pathways involved in different adverse environmental conditions such as stomatal closure, osmotic adjustment, and the activation of defense mechanisms [69,70]. These pathways may include those mediated by phytohormones such as abscisic acid (ABA), jasmonic acid (JA), and salicylic acid (SA), as well as calcium signaling, reactive oxygen species (ROS) signaling, and various stress-responsive gene networks [71,72]. When ROS are generated within plant cells, they lead to oxidative damage. Serotonin helps activate antioxidant defense systems, such as enzymes like superoxide dismutase (SOD), catalase (CAT), peroxidases, and glutathione-related enzymes [73]. These antioxidants scavenge ROS and protect the cellular components from oxidative stress, thereby enhancing plants’ tolerance to stress. Additionally, serotonin influences the ion transport processes across cellular membranes, helping maintain ion homeostasis under stress conditions [68]. This includes the regulation of the ion channels, transporters, and pumps involved in the uptake, efflux, and compartmentalization of ions such as potassium (K^+^), sodium (Na^+^), calcium (Ca^2+^), and magnesium (Mg^2+^). Proper ion homeostasis is crucial for maintaining cellular turgor pressure, osmotic balance, and enzyme activities during stress [74]. When abiotic stress leads to metabolic reprogramming in plants, involving shifts in the primary and secondary metabolite profiles to adapt to changing environmental conditions, serotonin acts as a key player in metabolic regulation, influencing the synthesis and accumulation of metabolites such as compatible solutes (e.g., proline, sugars, polyols) and secondary metabolites (e.g., phenolic compounds, including flavonoids) to sustain essential physiological processes under stress.

Moreover, serotonin plays a vital role in fine-tuning plants’ responses to different abiotic stressors by triggering the activation of stress tolerance mechanisms that include the activation of transcription factors and the upregulation of genes encoding stress-related proteins, enzymes involved in antioxidant defense, osmotic adjustments, and ion transporters that regulate ion homeostasis under stress conditions [75]. Since abiotic stress often inhibits plants’ growth and development, serotonin can modulate these processes by influencing hormone signaling cascades and gene expression networks associated with growth regulation [76]. It may promote adaptive growth responses or allocate resources to stress tolerance mechanisms, depending on the severity and duration of the stress. Scientific evidence suggests that serotonin may also exert epigenetic effects on gene expression in response to abiotic stress. Epigenetic modifications, such as DNA methylation, histone modifications, and small RNA-mediated gene silencing, can influence chromatin accessibility and regulate the expression of stress-responsive genes [77]. Serotonin may interact with epigenetic regulators to modulate the chromatin structure and gene expression patterns, providing an additional layer of complexity to its regulatory role in stress responses [78]. Further, serotonin is likely engaged in a network of cross-talk and feedback loops with other signaling molecules and pathways involved in stress responses [68]. This allows for the integration of multiple stress signals and the coordination of adaptive responses. Cross-talk between serotonin and other signaling molecules, such as nitric oxide (NO), reactive nitrogen species (RNS), and phytohormones, enhances the complexity and robustness of adaptive plant stress responses [79]. Equally, the plant microbiota, including beneficial microbes such as rhizobacteria and mycorrhizal fungi, play important roles in modulating plants’ stress responses and enhancing their stress tolerance. Serotonin may interact with the plant microbiota, influencing microbial community dynamics and function [80]. Conversely, the microbial metabolites and signaling molecules produced in response to stress may provide feedback to regulate serotonin biosynthesis and signaling in plants, forming complex cross-kingdom interactions that contribute to stress adaptations [62].

It is worth to mention that serotonin-mediated stress responses may also be transmitted across generations through epigenetic mechanisms, allowing plants to retain memory of past stress exposures and prime their offspring for enhanced stress tolerance [81]. This transgenerational memory enables plants to anticipate recurring stress events and adapt more rapidly to environmental changes over successive generations [81,82].

Given its central regulatory role, genetic engineering approaches to enhancing serotonin biosynthesis or the application of exogenous serotonin or serotonin precursors can be harnessed to develop resilient stress-tolerant crop varieties and sustainable agricultural practices.

## 5. Serotonin Activates Antioxidant Defense Systems in Response to Cd Stress

A major mechanism through which cadmium causes toxicity is by inducing oxidative stress [4]. Plants possess a sophisticated antioxidant defense system to counteract the damaging consequences of ROS and maintain cellular redox homeostasis [15].

### 5.1. The Role of Serotonin and Its Mechanisms of Action

Serotonin plays a role in activating antioxidant defense systems in response to cadmium stress. The precise mechanisms underlying serotonin-mediated activation of the antioxidant defense systems in response to cadmium stress are still being elucidated [83]. Studies have shown that the serotonin levels increase in plants upon exposure to cadmium, suggesting its involvement in plants’ stress response mechanisms [84]. It is believed that serotonin may interact with specific receptors or signaling pathways involved in stress perception and transduction, activating downstream stress-responsive genes and antioxidant enzymes. Serotonin may also modulate cellular redox signaling pathways and transcription factors that regulate antioxidant gene expression [71,85]. Furthermore, serotonin may indirectly influence the antioxidant defense systems by modulating the activity of other stress-responsive molecules or secondary messengers involved in ROS signaling [71,85]. For example, serotonin can directly enhance the expression of genes encoding activated antioxidant enzymes such as superoxide dismutase (SOD), catalase (CAT), peroxidase (POX), and ascorbate peroxidase (APX), thereby increasing their activity levels and enhancing the ROS scavenging capacity [73]. Additionally, serotonin may stimulate the synthesis of non-enzymatic antioxidants such as ascorbate (vitamin C), tocopherols (vitamin E), and phenolic compounds, enhancing a plant’s ability to detoxify ROS and protect the cellular components from oxidative damage [86]. By activating the antioxidant defense systems, serotonin enhances a plant’s ability to withstand cadmium stress and mitigate oxidative damage. Plants with higher levels of serotonin or enhanced serotonin biosynthesis are often more tolerant to cadmium toxicity, exhibiting reduced oxidative stress, a boosted enzymatic antioxidant defense system, and a better growth performance under cadmium-contaminated conditions. Therefore, manipulating serotonin levels or its signaling pathways holds potential for maintaining cellular homeostasis, improving plants’ tolerance to cadmium stress, and enhancing the phytoremediation strategies for contaminated soils.

### 5.2. Transcriptional Regulation

Serotonin regulates the expression of genes encoding antioxidant enzymes and other stress-responsive proteins through transcriptional regulation [71,85]. It acts on specific transcription factors or regulatory elements in the promoter regions of target genes, thereby modulating their transcriptional activity [87]. For example, serotonin may activate transcription factors such as NAC (NAM, ATAF1/2, CUC2) [88] and AP2/ERF (APETALA2/ethylene-responsive factor) [89] family proteins, which regulate the expression of stress-responsive genes, including those encoding antioxidant enzymes. This transcriptional reprogramming orchestrated by serotonin could enhance a plant’s ability to withstand Cd stress [90].

### 5.3. Post-Translational Modifications

In addition to transcriptional regulation, serotonin may exert its effects on the antioxidant defense systems through the post-translational modification of proteins. Hydrogen sulfide (H_2_S) was previously thought to be a dangerous gaseous molecule in plant cells. However, this idea has now been altered as a result of plants using H_2_S in several developmental processes [91,92,93]. It has an impact on several growth and development traits at reduced concentrations, such as germination rates [94], stomatal control [95], and adventitious root development [93]. Furthermore, through persulfidation (S-sulfhydration), H_2_S can modify proteins post-translationally, changing their functional consequences and locations. To regulate stress, growth, and development, H_2_S interacts significantly with phytohormones like auxin, serotonin, melatonin, and abscisic acid [96,97,98]. Plants require serotonin, which is converted into melatonin, to maintain H_2_S equilibrium. Melatonin influences the production of cytosolic L-DES isoforms, which helps the cells respond to stress by preserving H_2_S equilibrium. When melatonin was used to pre-treat cucumbers, L/D-cysteine desulfhydrase was stimulated to produce H_2_S, which affected the photosynthetic efficiency and ROS burst [99]. H_2_S interacts with NO and MAPK pathways to act as a downstream target of melatonin when it is employed to reduce stress.

### 5.4. The Role of Serotonin Transporters

Serotonin transporters are integral membrane proteins that facilitate the uptake of serotonin into plant cells. These transporters play a crucial role in regulating serotonin levels and signaling dynamics in response to environmental stimuli. Although direct evidence on the role of plant serotonin transporters in Cd stress is limited, the current findings suggest that the ectopic expression of MmSERT, a serotonin transporter gene, in transgenic apple and Arabidopsis showed improved salt tolerance, reduced reactive oxygen species (ROS) production, and increased melatonin levels, suggesting the potential role of serotonin transporters in stress response mechanisms [100].

Serotonin and auxin show structural resemblances that convey the promise of auxin receptors as surrogates for serotonin transport in plants [101]. Understanding the regulation of the serotonin transporters and their impact on serotonin-mediated antioxidant responses could provide further insights into the molecular mechanisms underlying plant stress tolerance.

### 5.5. The Integration of Hormonal Signaling with Growth and Development

Young leaves have a noticeably low serotonin content; however, senescence, pathogen infection, and nutritional shortages cause plants to experience an abrupt increase in serotonin [102,103]. Serotonin accumulates in high concentrations in senescent tissues, and its significant antioxidant activity in the leaves demonstrates its ability to slow down senescence. Since serotonin is the precursor of melatonin, it is more easily induced by infections, aging, and environmental stressors. N-acetylserotonin deacetylase (ASDAC) and serotonin N-acetyltransferase (SNAT) are involved in a reversible melatonin production pathway. Melatonin production is stimulated by SNAT and inhibited by ASDAC, suggesting that these two enzymes closely regulate the amount of melatonin in plants to keep it at an ideal level [104]. Serotonin controls plants’ growth, development, and morphogenesis in addition to its function in stress resistance. By controlling auxin signaling, serotonin enhances shoot organogenesis from root cultures [105,106]. According to Erland, Turi, and Saxena [62], melatonin and serotonin worked with their metabolites to control morphogenesis in *Hypericum perforatum* explants in vitro. It is well known that root organogenesis in vitro is primarily regulated by the relative ratio of auxin to cytokinin. The results of Murch et al. [105] on *Hypericum perforatum* explants pointed to a proposed role of an alternate metabolic pathway originating with tryptophan, more specifically the relative ratios of serotonin, melatonin, and IAA, in the regulation of plant morphogenesis. This cascade of phytochemical responses integrates several pathways and the phytohormone network, including ethylene, auxin, cytokinin, abscisic acid (ABA), and salicylic acid signaling [62].

It is possible that these research results can explain the role of serotonin’s interaction with phytohormone signaling pathways to coordinate growth and biomass accumulation and plant responses to Cd stress. The cross-talk between serotonin and hormonal signaling networks modulates antioxidant defense systems and other stress-responsive pathways, enabling plants to mount effective adaptive responses. Elucidating the molecular mechanisms underlying the hormonal regulation of serotonin-mediated antioxidant responses and the maintenance of cellular functions would explicate the complex interplay between different signaling pathways in Cd stress tolerance.

### 5.6. Mitochondrial Protection

Cd has been purported to inhibit the mitochondrial electron transport chain (ETC) by impairing electron flow. This hardens mitochondrial function and leads to the generation of ROS within the organelle [107]. By preserving mitochondrial function, serotonin ensures efficient energy production and cellular metabolism under Cd stress [108]. In a study on perennial ryegrass, the Cd-treated plants displayed higher levels of malondialdehyde and peroxidase (POD), catalase, and superoxide dismutase (SOD) activities compared to those in the control. Cd stress stimulated upregulation of the expression of FeSOD, MnSOD, ChlCu/ZnSOD, Cyt Cu/ZnSOD, APX, GPX, GR, and POD at 4–24 h following the start of the treatment. It was suggested that this gene transcript profile was related to the enzyme activity under Cd stress [108]. Thus, serotonin helps protect the mitochondria from Cd-induced damage by enhancing antioxidant defense systems localized within the organelle.

### 5.7. The Interplay with the Secondary Metabolism

Secondary metabolites are organic substances that are non-essential to the basic functions of plants, but they play important roles in their adaptation, defense, and communication [109]. Cadmium can affect the levels and activities of secondary metabolites which are involved in the detoxification and protection of plants. Cd can also activate the expression of genes and enzymes related to the biosynthesis of secondary metabolites, such as phenylalanine ammonia-lyase, chalcone synthase, and flavonoid 3′ hydroxylase [110]. For example, Cd induced the accumulation of flavonoids and anthocyanin in *Arabidopsis thaliana* and increased the production of glucosinolates and camalexin in *Brassica juncea* [110].

Serotonin biosynthesis is interconnected with the metabolism of secondary compounds such as flavonoids, phenolic acids, and alkaloids, which possess antioxidant properties depending on the plant species and the environmental conditions, such as Cd stress, contributing to the overall antioxidant capacity of plants. For example, serotonin can increase the production of indole alkaloids in *Catharanthus aoseus* and Vinblastine in *Vinca minor* [111].

### 5.8. Genetic Variation and Serotonin Regulation

Genetic variation among plant species and cultivars can influence serotonin biosynthesis and signaling pathways, affecting their susceptibility to Cd stress. Natural genetic variations in serotonin-related genes may influence how plants cope with cadmium stress. For example, comparative and functional metabolomic investigations were performed under cadmium stress to test the metabolic basis of this characteristic in *Brassica napus* [112]. The metabolomic reactions of the tolerant Cd-accumulating genotype (CB671) and its sensitive counterpart (ZD622) were shown to be both conserved and differential. In response to Cd stress, CB671 redirected the carbon flux to produce ascorbate, sugar storage forms, jasmonates, ethylene, and vitamin B6—all suitable solutes. Interestingly, there was a 1.91-fold decrease in the abundance of IAA, which was associated with a 3.48-fold increase in the derivation of serotonin from tryptophan. In contrast, Cd caused a significant reduction in vitamins and carbs in ZD622, but it also slightly altered hormone levels. A notable build-up of oxylipins and unsaturated fatty acids in CB671, along with an increase in glycerophospholipids and the stimulation of signaling metabolites produced from inositol, revealed the capacity to initiate detoxification processes quickly [112]. Studying the genetic variation in serotonin-related genes and their association with Cd tolerance traits may identify candidate genes for genetic improvement programs developing stress-tolerant crop varieties.

## 6. Conclusions and Remarks

By coordinating a multifaceted response that encompasses the antioxidant defense systems, transcriptional regulation, enzymatic activation, post-translational modifications, and interplay with the secondary metabolism, serotonin serves as a master regulator of the antioxidant defense systems in plants under Cd stress. This comprehensive regulatory network ensures the effective scavenging of ROS; the maintenance of cellular redox homeostasis, integrity, and function; and ultimately the promotion of plants’ survival and adaptation to Cd-contaminated environments.

Future studies should focus more deeply on the molecular processes and signaling networks that serotonin uses to exert its regulatory functions. Integrating omics technologies, including proteomics, metabolomics, and transcriptomics, may reveal new serotonin-responsive genes and pathways essential for adapting to metal stress.

Furthermore, knowing how serotonin interacts with secondary metabolites and phytohormones may help identify synergistic processes that increase resilience to stress. These discoveries may open the door for biotechnological approaches such as metabolic priming or genetic engineering to increasing the serotonin production or signaling in crops.

Moreover, researchers need to gain a more comprehensive understanding and knowledge to guide the development of innovative strategies for enhancing crop productivity and environmental sustainability in regions affected by heavy metal pollution.

## Data Availability

All of the data are presented in the review.

## Abbreviations

The following abbreviations are used in this manuscript:

Cd	cadmium
Ser	serotonin
Pb	lead
Hg	mercury
As	arsenic
RuBP	ribulose-1,5-bisphosphate
ROS	reactive oxygen species
ER	endoplasmic reticulum
UPR	unfolded protein response
PAL	phenylalanine ammonialyase
POD	peroxidase
4CL	4-coumarate CoA ligase
COMT	caffeic acid 3-O-methyl transferase
CAD	cinnamyl alcohol dehydrogenase
CCoAOMT	cafeoy1-CoA3-O-methy1transferase
PCs	phytochelatins
MTs	metallothioneins
HMA	heavy metal ATPase
NRAMPS	Natural Resistance-Associated Macrophage Proteins
CAX	cation exchangers
SNAT	acyl-CoA-dependent serotonin N-acyltransferase
ASMT	N-acetylserotonin methyltransferase
ABA	abscisic acid
JA	jasmonic acid
SA	salicylic acid
SOD	superoxide dismutase
CAT	catalase
POX	peroxidase
APX	ascorbate peroxidase
ASDAC	N-acetylserotonin deacetylase
SNAT	serotonin N-acetyltransferase
ETC	electron transport chain
